# Herpes‐like skin lesion after AstraZeneca vaccination for COVID‐19: A case report

**DOI:** 10.1002/ccr3.4883

**Published:** 2021-10-04

**Authors:** Mohammadreza Ardalan, Hamidreza Moslemi, Shervin Shafiei, Reza Tabrizi, Mohammadreza Moselmi

**Affiliations:** ^1^ Kidney Research Center Tabriz University of Medical Sciences Tabriz Iran; ^2^ Postgraduate Student of Oral and Maxillofacial Surgery Department of Oral and Maxillofacial Surgery Dental School Shahid Beheshti University of Medical Sciences Tehran Iran; ^3^ Department of Oral and Maxillofacial Surgery Dental School Shahid Beheshti University of Medical Sciences Tehran Iran; ^4^ Postgraduate Student of Internal Medicine Department of Internal Medicine School of Medicine Tabriz University of medical sciences Tabriz Iran

**Keywords:** Chadox1 ncov‐19 vaccine, COVID‐19, COVID‐19 vaccines, herpes simplex, varicella zoster

## Abstract

Recurrent herpes simplex virus or varicella zoster virus infection should be considered as one of the rare complications after AstraZeneca vaccination for COVID‐19.

## Graphical abstract

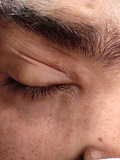

## 1 INTRODUCTION

Vaccination against coronavirus disease 2019 (COVID‐19) has been launched by many countries. Since then, viral infections after vaccination have been reported. This study reports a case of herpes‐like skin lesions after chadox1 ncov‐19 (azd1222) vaccine in a 28‐year‐old man and reveals a possible relation between this vaccine and viral reactivation.

Since coronavirus disease 2019 (COVID‐19), pandemic, safe, and effective vaccination against it became vital. Up to now, more than 50 vaccines against severe acute respiratory syndrome coronavirus 2 (SARS‐CoV‐2) have been developed and presented in clinical trials. Chadox1 ncov‐19 (AZD1222) is one of them, which has been used in the national vaccination programs against the disease in many countries.^1^ Viral infections after COVID‐19 vaccination have been reported. Furer et al, have reported six cases of herpes zoster following BNT162b2 mRNA COVID‐19 vaccination.^2^

Herpes virus reactivation has been reported following trivalent influenza, hepatitis A, and rabies vaccines, suggesting vaccine‐modulated immunomodulation.^3^ To our knowledge, herpes‐like skin lesions have not been reported in the azd1222 vaccine clinical trials,^4^ but some spontaneous reports in this regard from the UK have been received between April 1 and July 21, 2021.

In this study, we are going to report a herpes‐like skin lesion following AZD1222 vaccination.

## 2 CASE PRESENTATION

**FIGURE 1:**
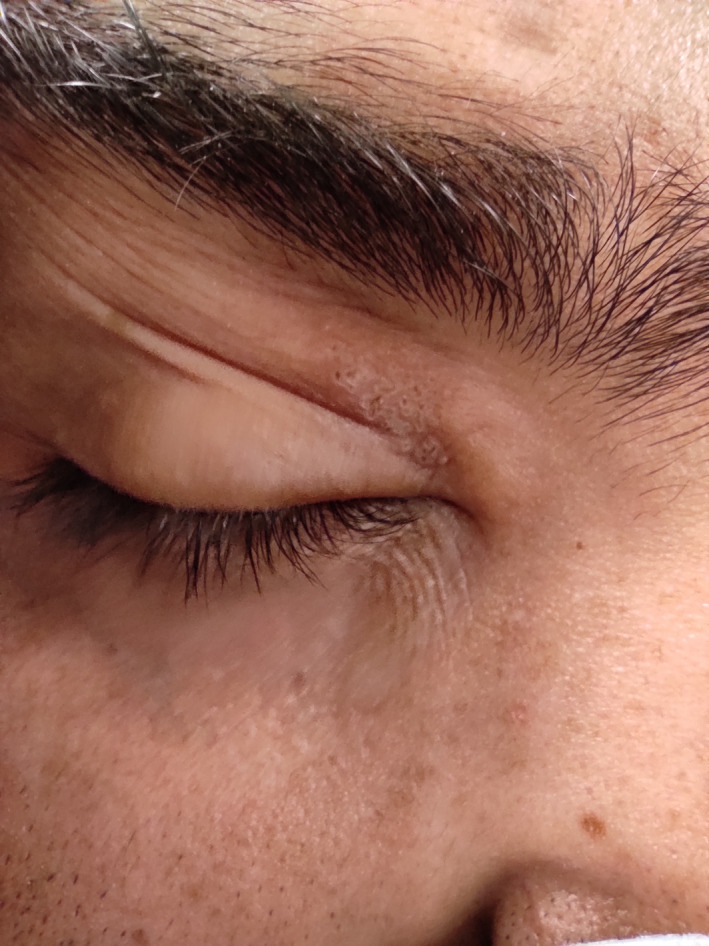
Blistery skin rashes developed on the right upper eyelid 2 days after vaccination with chadox1 ncov‐19 (AZD1222)

**FIGURE 2:**
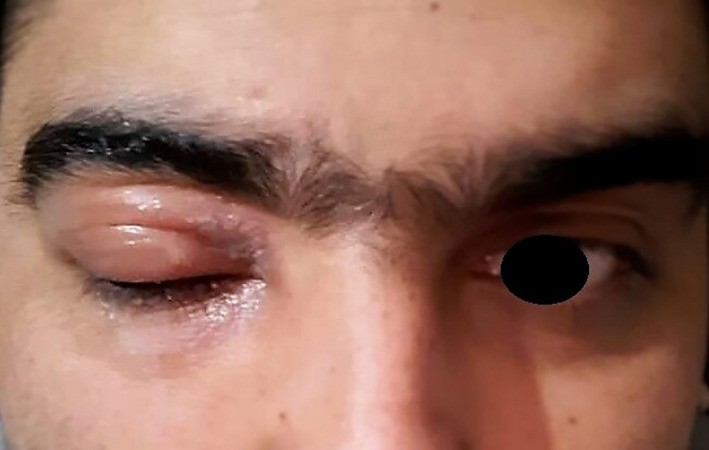
Edema of the upper eyelid 5 days after vaccination with chadox1 ncov‐19 (AZD1222)

A 28‐year‐old man, with no systemic disease, presented with herpes‐like skin lesions on his right upper eyelid. He received first dose AZD1222 vaccine on March 20, 2021, during the Iran national vaccination program. Besides the common post‐vaccination signs and symptoms, 2 days later, burning sensation, and painful skin rashes, and blisters developed on his right upper eyelid (Figure 1) followed by upper eyelid edema 3 days later (Figure 2). Ocular examination was done by slit‐lamp, which showed normal findings. Along with these findings, patient reported a history of a herpes simplex virus (HSV) infection following a trauma to the right eye with an object in his childhood. According to the patient, he has experienced periods of cold sore (herpes simplex labialis) ever since. Regarding clinical signs and symptoms and patient history, treatment was started based on the diagnosis of recurrent HSV lesion of the upper eyelid. Topical antibiotic therapy was administered for the patient including sulfacetamide 10% eye drop to treat probable bacterial superimposition and conjunctivitis and blepharitis because of severe periorbital edema and ketotifen eye drop, Vitamin A ointment to reduce itching and irritation. After 72 h, the patient reported blurred vision on the right eye. Ocular examinations were normal again. Seven days after the onset of the lesion, signs and symptoms disappeared, and treatments were discontinued.

## 3 DISCUSSION

We reported a 28‐year‐old man who developed herpes‐like skin lesions as vesicular ulcerative periorbital lesion 48 h after the first dose of AZD1222 vaccine against COVID‐19.

Since the onset of COVID‐19 pandemic, herpes simplex, or herpes zoster‐like lesions have been reported during COVID‐19 infection.^5^, ^6^ This might be explained through the function of natural killer group 2D (NKG2D) ligands which are also known as “stress‐induced ligands”, found on healthy cells, such as neuronal cells, in order to avoid auto‐reactivity of natural killer T cells against normal tissues. HSV and varicella zoster virus (VZV) cause downregulation of NKG2D ligands on neural cell membranes and terminals after infection of these cells, so the infected cells could not be recognized with natural killer cells, and the HSV or VZV infection enters its latent phase.^7^, ^8^ These ligands, however, are upregulated on the cell surface following various stresses including oncogene activation, hypoxia or viral infections,^9^, ^10^ and the last two conditions are present in COVID‐19 which can explain the coincidence of COVID‐19 infection and HSV or VZV reactivation.

Also, AZD1222 is a replication‐deficient simian adenoviral vector that expresses the full‐length SARS‐CoV‐2 spike protein. Ewer et al.^11^ showed that this vaccine can initiate cytokine release and immune response cascades in a similar way to viral infections and SARS‐CoV‐2. This mechanism can lead to upregulation of NKG2D ligands and reactivation of the HSV or VZV from the latent phase and development of the clinical signs and symptoms of herpes‐like skin lesions after vaccination.

The diagnosis of mucocutaneous involvement of HSV and its differentiation from herpes zoster infection was based on clinical signs and one of the limitations of the present report. Serologic and molecular virus detection tests should have been done for definite diagnosis.

## 4 CONCLUSION

This study revealed a possible relation between AZD1222 vaccination and HSV or VZV reactivation, which also cannot be proved based on this report alone. Awareness should be raised regarding the potential link between COVID‐19 infection or vaccination and HSV or VZV reactivation. Further similar reports and safety monitoring studies on AZD1222, and other COVID‐19 vaccines’ side effects are required for a more powerful conclusion.

## CONFLICT OF INTEREST

None.

## AUTHOR CONTRIBUTIONS

MA and MM did the conception and study design. HM and SS drafted the article or critically revised it for important intellectual content. RT and MM approved the final submitted version.

## ETHICAL APPROVAL

This study was performed according to the principles outlined by the World Medical Association's Declaration of Helsinki on experimentation involving human subjects, as revised in 2000 and has been approved by the ethics committee of the Tabriz University of Medical Sciences.

## CONSENT

Written informed consent was obtained from the patient for the publication of this report and clinical images.

## Data Availability

The data that support the findings of this study are available from the corresponding author upon reasonable request.
